# Infections @ Trauma/Orthopedic Implants: Recent Advances on Materials, Methods, and Microbes—A Mini-Review

**DOI:** 10.3390/ma14195834

**Published:** 2021-10-06

**Authors:** Britt Wildemann, Klaus D. Jandt

**Affiliations:** 1 Experimental Trauma Surgery, Department of Trauma, Hand and Reconstructive Surgery, Jena University Hospital, Friedrich Schiller University Jena, 07747 Jena, Germany; 2 Chair of Materials Science, Otto Schott Institute of Materials Research, Faculty of Physics and Astronomy, Friedrich Schiller University, 07743 Jena, Germany; k.Jandt@uni-jena.de

**Keywords:** mini-review, orthopedics, biomaterial associated infection (BAI), *Staphylococcus aureus*, surface structuring, drug delivery, antimicrobial, implant materials

## Abstract

Implants and materials are indispensable in trauma and orthopedic surgery. The continuous improvements of implant design have resulted in an optimized mechanical function that supports tissue healing and restoration of function. One of the still unsolved problems with using implants and materials is infection. Trauma and material implantation change the local inflammatory situation and enable bacterial survival and material colonization. The main pathogen in orthopedic infections is *Staphylococcus aureus*. The research efforts to optimize antimicrobial surfaces and to develop new anti-infective strategies are enormous. This mini-review focuses on the publications from 2021 with the keywords *S. aureus* AND (surface modification OR drug delivery) AND (orthopedics OR trauma) AND (implants OR nails OR devices). The PubMed search yielded 16 original publications and two reviews. The original papers reported the development and testing of anti-infective surfaces and materials: five studies described an implant surface modification, three developed an implant coating for local antibiotic release, the combination of both is reported in three papers, while five publications are on antibacterial materials but not metallic implants. One review is a systematic review on the prevention of stainless-steel implant-associated infections, the other addressed the possibilities of mixed oxide nanotubes. The complexity of the approaches differs and six of them showed efficacy in animal studies.

## 1. Introduction

Due to the advances in orthopedic and trauma-care, such as modern imaging methods, improved surgical techniques, and optimized design of implants and materials, patients are successfully treated [1]. Despite these advances, infection is still a potential complication and often difficult to treat [2,3]. Infections in orthopedic and trauma surgery include surgical site infections (SSI), periprosthetic joint infections (PJI), fracture-related infections (FRI), and biomaterial- or implant-associated infections (BAI or IAI). The main pathogen responsible for BAIs is *Staphylococcus aureus* (*S. aureus*) [2,4]. *S. aureus* are Gram-positive bacteria, which can be methicillin-sensitive (MSSA) or methicillin-resistant (MRSA). They produce virulence factors to interact with proteins of the host extracellular matrix (ECM) (microbial surface components recognizing adhesive matrix molecules, MSCRAMMs), factors facilitating ECM breakdown, and host cytotoxic factors such as phenol-soluble modulins (PSMs). Planktonic bacteria can adhere to surfaces and form a biofilm that protects the bacteria from the immune system and antibiotics. The metabolically less active, less virulent, and slow growing small colony variants (SCV) of *S. aureus* occur often in biofilms. *S. aureus* can also invade host cells and persist there for a prolonged time period. More detailed information on the role of *S. aureus* in osteomyelitis can be found in the review by Nasser et al. [5]. 

Although the rate of PJI is low, it has a dramatic effect on the patients’ health; it is difficult to eradicate resulting in a severe complication with a significant patient and socioeconomic burden [6].

The risk for FRI depends on the severity of the trauma and the associated tissue damage and is reported as up to 31% in open fractures treated systemically with antibiotics and can be reduced to 9% with additional local antibiotics [7].

The incidence for BAI ranges from 0.1% (intraocular lenses) up to 33% (urinary tract catheter), with 1–7% for prostheses or nails [8]. This is due to the attractiveness of material surfaces to bacteria and the impaired host defense in injured tissue [9,10].

Clinically, local antibiotic treatment is via topical antibiotic application [11], antibiotic loaded poly(methyl-methacrylate), which must be removed [12], or by silver, povidone-iodine, or antibiotic coatings of the implant [13,14,15]. The coating of implants is an attractive approach because implanted materials increase the risk of infection. Therefore, the optimization of material surfaces or the development of entirely new antimicrobial materials concepts is of great interest to reduce the infection risk. The research on implant or surface modifications, coatings, antimicrobial substances, and combinations thereof has increased over the last decades. The translation into clinical application, however, is still limited due to the often very complex modification methods and demanding regulatory requirements [16]. This review summarizes the latest research on anti-infective strategies in the field of orthopedics and trauma surgery focusing on materials, their modifications, and the most relevant microbe: *S. aureus*.

## 2. Materials and Methods

A PubMed-search was performed using the following BOOLEAN operators: *S. aureus* AND (surface modification OR drug delivery) AND (orthopedics OR trauma) AND (implants OR nails OR devices) on 5 July 2021. This research resulted in 415 publications, the first published in 1992 (Table 1). To narrow this review to the most recent publications, the search was limited to the year 2021, resulting in 18 publications. All 18 publications were included in this mini-review as they all match the search criteria and were published in English without duplicates.

## 3. Results

The search strategy resulted in a total of 415 publications with the first published 1992 (Table 1). In 2021 (until July), a total of 18 scientific papers were published: 16 original studies and 2 reviews. As can be seen from the table, a strong increase can be observed with an approximate doubling of the number of publications for each period.

### 3.1. Original Publications

The 16 original studies can be grouped into 1. surface modifications, 2. local antibiotic release, 3. combination of both, and 4. non-metallic material modifications (Table 2).

#### 3.1.1. Surface Modifications

**Figure 1:**
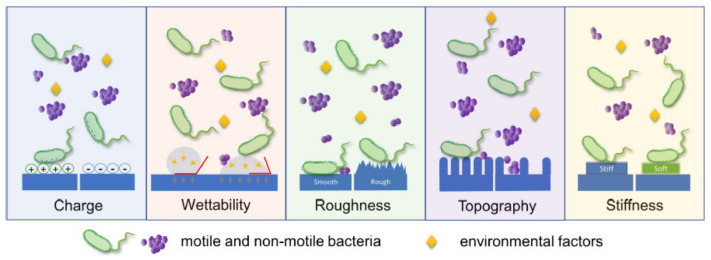
Materials’ surface characteristics that influence the adhesion of microbes. The figure is taken from the publication from Zeng et al. [33] in accordance with copyright permission guidelines of the journal, CC BY 4.0.

Surface characteristics such as charge, wettability, roughness, topography, stiffness, and ion release have a major influence on the adhesion of bacteria (Figure 1) [33], which was the topic of five publications.

The easiest approach to translate in clinical applications would be pure surface modification without coating or the addition of antimicrobial substances. This approach was chosen by Meinshausen et al. using a periodic line-like surface structure to affect bacterial adhesion [17]. Structuring titanium (Ti) surface by laser interference patterning or polyethylene terephthalate (PET) surfaces by roll-to-roll hot embossing revealed a clear effect of the aspect ratio on the adhesion of *S. aureus* with the most prominent effect at 0.02 to 0.05. This can be explained through different ways in which the bacteria interact with the substrate, such as molecular interactions, free surface energy, or hydrophobicity [9].

More complex approaches use coatings or the addition of other substances. In one study, the effect of surface modifications on the formation of a difficult to eradicate biofilm was investigated [18]. The coating of Ti alloy with pure Ti, silver (Ag), hydroxyapatite (HA), or tricalcium phosphate (TCP) as well as the effect of rough blasting (rb) were analyzed. With comparable cell content on all surfaces, biofilms grew less strongly on smoother surfaces (base Ti alloy or nitride coated Ti) compared to rougher surfaces such as the rb, HA, and TCP surfaces, which was also shown by the expression of biofilm-associated genes. Silver coating had no clear inhibiting effect on biofilm formation.

An optimized surface structure and modification might not only reduce bacterial adhesion but might, in contrast, be attractive for osteoblasts and therefore stimulate osteointegration. This approach was followed by the development of a modular hybrid biocoating based on the modification of porous Ti with polydopamine (PDA), ZnO nanoparticles (NP), and chitosan (CS)/nano HA [19]. PDA and ZnO resulted in, e.g., a reduced wettability, protein absorption, and bacterial adhesion. Compared to pure titanium, the surface modifications reduced the viability of MC3T3 cells, most pronounced with PDA-nZnO. Interestingly, the addition of CS/nHA improved the biocompatibility and osteogenic differentiation of the cells as demonstrated by increased alkaline phosphate activity.

**Figure 2:**
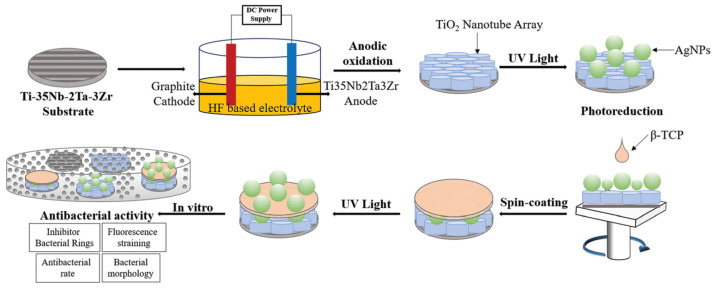
Surface modification of Ti–35Nb–2Ta–3Zr with nanotubes, AgNP, and TCP. Reproduced from Ref. [20] with permission from the Royal Society of Chemistry (doi:10.1039/d1nr02459k).

Ti alloys differ in their elastic moduli and Ti–35Nb–2Ta–3Zr has a lower modulus than the usually used Ti–6Al–4V, which is therefore more similar in modulus to bone (approximately 20 GPa). To further optimize the antimicrobial and osteoconductive properties of Ti–35Nb–2Ta–3Zr, the surface was structured with titanium nanotubes (NT) loaded with Ag nanoparticles (Figure 2) [20]. The silver was released over at least 28 days. All Ag-modifications inhibited *S. aureus* and *E. coli* growth. No negative effects of the leaching solutions from the surface modifications and the released Ag on mesenchymal stromal cells (MSC) were observed.

A metal-organic framework was used in the last study. Only the combination of sulfonated poly(etheretherketone) (PEEK) as 3D porous implant material functionalized with Ag loaded to the zinc-based zeolitic imidazolate framework-8 (ZIF-8) successfully reduced bacterial adhesion compared to the single modifications [21]. Neither the surface modifications nor the released zinc or silver affected the viability of L929 cells. The ZIF-8 complex as well as zinc and Ag ions were released from the modified surface. The authors highlighted the simplicity of the presented functionalization.

None of the developed surface modifications were tested in an in vivo preclinical study. The studies characterized the surface properties and the effect on bacteria and partially also on osteoblast-like cells or MSCs, but in vivo studies are important to show the efficacy of the approaches.

#### 3.1.2. Local Antibiotic Release

The combination of an implant with an antibiotic turns the stabilizing implant into a drug-eluting device. This approach opens the possibilities of anti-infective pharmaceutical approaches but makes the regulation of the combination device more challenging. Three studies developed an implant coating for local antibiotic release using different approaches.

Soylu et al. loaded a hydrogel with gentamicin to coat dopamine-functionalized titanium implants [22]. The agarose gel was crosslinked with tannic acid (TA) and calcium chloride (CaCl). Gentamicin was released over 14 days with a peak at 6 h. The addition of TA resulted in a delayed release—less burst but longer release—which was also confirmed by the inhibition of *S. aureus* growth. The TA-modified gel, however, inhibited cell viability (Saos-2 cells). The negative effects of TA on the cells were rescued by the additional application of CaCl.

The coating from Yu et al. is produced in a layer-by-layer technique with montmorillonite (MMT), poly-L-lysine (PLL), and vancomycin [23]. PLL can be hydrolyzed by chymotrypsin, which might be increased in infected tissue, or by *S. aureus* resulting in a triggered vancomycin release as demonstrated by the microbiological experiments. Viability of primary human osteoblast-like cells was not affected by the coating. The group also performed an animal study. Coated or uncoated k-wires were implanted in rat tibiae and *S. aureus* was inoculated. After sacrifice, reduced bacterial growth was detected on the coated implants and increased bone formation.

**Figure 3:**
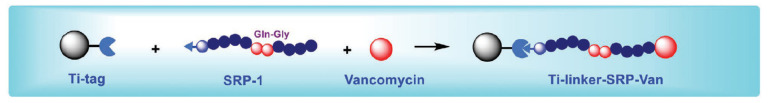
Schematic presentation of the infection-responsive coating. The Gln-Gly site (red double elements) of the SRP protein can be cleaved by the *S. aureus* protein SplB. Reproduced from Ref. [24] with permission from Wiley-VCH GmbH.

The third study also describes a coating with an infection-triggered release [24]. Vancomycin was conjugated with the SRP protein, which is cleavable by the *S. aureus* protein SplB then releasing vancomycin. Using click-chemistry, the SRP-vancomycin complex was attached to the titanium surface (Figure 3). Cleavage of vancomycin by SplB from the coated titanium was shown, as well as the specificity of the SRP protein to SplB cleavage. Cytocompatibility of the coating was demonstrated with an osteoblast cell line (MC3T3) and antimicrobial activity with *S. aureus*. The killing of microbes was increased after adding SplB.

#### 3.1.3. Surface Modifications and Local Antibiotic Release

The combination of surface structuring and local antibiotic release is an attractive approach and three studies were published on this topic.

In the first study, completely regular Ti nanotubes (TNT) were loaded with vancomycin (VA), modified with reduced graphene oxide (RGO), and coated with CS nanofibers [25]. RGO is expected to have antibacterial and pro-osteogenic effects. The modifications resulted in a prolonged VA release compared to pure TI. Testing the modifications without VA revealed an improved viability of MG63 cells and reduced bacterial viability and adhesion. The loading with VA increased the killing of *S. aureus*.

**Figure 4:**
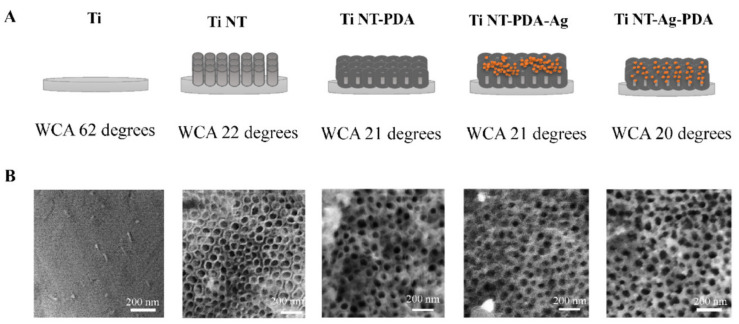
Schematic presentation (**A**) and electron microscopic pictures (**B**) of the different surfaces. WCA: water contact angle. Reproduced from Ref. [26] with permission from Elsevier.

The race for the surface [9] (i.e., that body cells occupy available surface sites on implants before microbes can adhere) won by host cells is important for the performance of an implant. Ren et al. used TNT loaded with AgNP incorporated in PDA (Figure 4) or gentamicin and investigated their effect on the coverage by human gingival fibroblasts and U2OS osteosarcoma cells, *S. aureus*, *P. aeruginosa*, or *S. epidermidis* and co-cultures [26]. PDA coating reduced the gentamicin load in the TNT due to reduced diameter, but both modifications showed a release over 48 h. Loading of TNT with gentamicin or AgNP killed the bacteria, except for *S. epidermidis* with no effect of gentamicin. The AgNP had negative effects on the adherence of host cells. The co-culture studies showed a better coverage with host cells when the TNT was loaded with gentamicin.

Based on a just recently characterized surface modification, the surface stability, effect on cells and bacteria, as well as osteointegration and antibacterial effectivity in vivo was evaluated [27]. Amphora-shaped pores of the Ti were coated with Ag and loaded with gentamicin. The surface was mechanically stable, cytocompatible, and anti-infective. Using a rat model, the surface modification showed an improved osteointegration without negative effect of the released gentamicin. In the rat infection model, the gentamicin-loaded implants significantly reduced the infection parameters and the bacterial contamination. Interesting for clinical application is the possibility to load the implant with the antibiotic during surgery, making the production and storage easier and allowing more flexibility.

#### 3.1.4. Modification of Non-Metal Implants

A group of the AO Research Institute, Davos, Switzerland, published two laborious studies using sheep infection models to test the efficacy of a gentamicin and vancomycin-loaded hydrogel. One study described the effect of a single-stage revision [28], while in the other study, a two-stage model was used [29]. Both studies used poly(N-isopropylacrylamide)-grafted thermoresponsive hyaluronic acid hydrogel loaded with gentamicin and vancomycin and systemic antibiotic treatment. In the single-stage study, the hydrogel plus the implant was inserted 8 weeks after infection with methicillin-resistant *S. aureus* (MRSA). In the two-stage revision, the hydrogel was applied 8 weeks after infection without a nail, which was inserted after further two weeks with an additional application of the hydrogel. The control received an antibiotic-laden bone cement-coated rod. Both studies showed an antibacterial effect of the hydrogel with a significantly better reduction of bacteria compared to the antibiotic laden bone cement in the two-stage revision. No difference was seen in the single-stage revision. The benefit of the hydrogel compared to the clinically approved bone cement is the degradation and the controlled antibiotic release.

A combined approach was pursued by Harrison et al. that aimed at the reduction of infection, especially the prevention of a biofilm and pain [30]. The biofilm preventing agent Cis-2-decenoic acid (C2DA) in combination with the anesthetic bupivacaine was mixed with electrospun CS membranes. The total release of the single drugs was dosage-dependent occurring in a sustained manner after an initial burst and slightly different to the release from the dual loaded membranes. The drug-loaded membranes were effective against planktonic bacteria and biofilm, however, bupivacaine inhibited L929 fibroblast viability.

**Figure 5:**
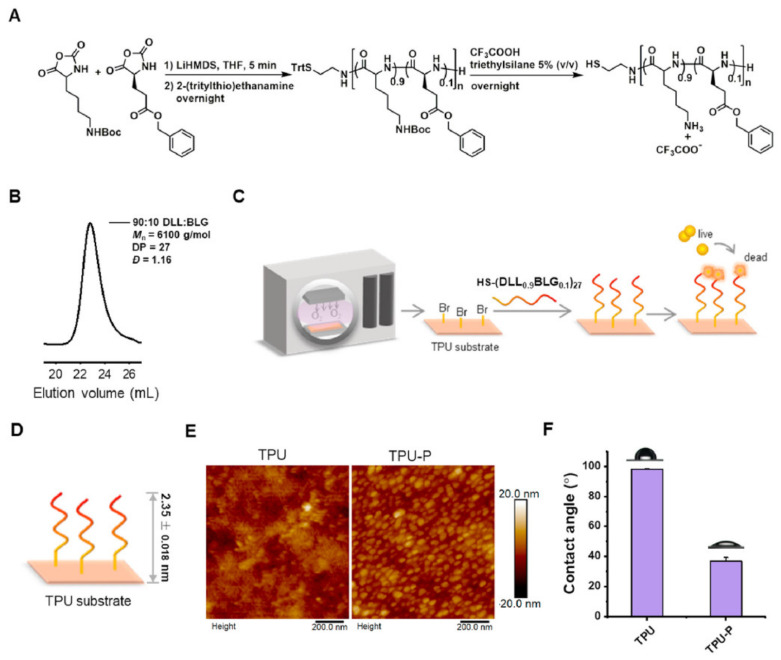
Preparation and analyses of the peptide modified TPU surface. (**A**) Synthesis of the polymer, (**B**) characteristics of the peptide polymer, (**C**) modification of the polymer surface, (**D**) thickness of the peptide layer on the TPU-substrate, (**E**) atomic force microscopic pictures of the TPU and peptide modified TPU surface, (**F**) water contact angles of the two different surfaces. Reproduced from Ref. [31].

A thermoplastic poly(urethane) (TPU) was modified with host defense peptide-mimicking peptide polymers and tested in vitro and in vivo (Figure 5) [31]. The peptide modified TPU revealed antimicrobial activity against different Gram-positive or -negative bacterial strains, with good hemocompatibility and without affecting HUVEC and 3T3 cells. Subcutaneous implantation of the peptide TPU in rats significantly reduced the bacterial colonization compared to TPU alone with good biocompatibility. The authors propose this peptide modified antibacterial surface as a promising platform technology to reduce IRI.

AgNP-containing viscose membranes with and without natural polymer coating were developed to support skin burn wound healing and reduce the infection risk [32]. The Ag-loaded viscose showed small inhibition zones of *S. aureus* around the material and an inhibiting effect on *E. coli*, which was reduced by the polymer coating. The polymer coating also affected strength, permeability, and swelling ratio of the viscose. In a rat skin burn wound model, all viscose modifications supported wound healing, while the best effect was seen with the clinical standard.

### 3.2. Reviews

The review and meta-analysis performed by Tsikopoulos et al. assessed the potential of stainless steel and its modifications to prevent *S. aureus* infection in animal models [34]. The analysis showed that passive or active coating of stainless-steel implants reduces MRSA or MSSA infections in various animal models using different antimicrobial substances. Interestingly, none of the 2021 published studies used stainless steel for orthopedic implants, but titanium.

The second review addressed the interesting question if nanotubes are relevant in nanomedicine with a special focus on mixed oxide nanotubes (MON). The review first introduced the concept of mono and mixed nanotubes and discussed the different mixed nano tubes depending on the material base. In the next part, detailed information on the in vitro properties of nanotubes on biocompatibility, biomineralization, antibacterial activity, and cellular processes was given. In the section on the in vivo studies, the authors summarized the results of different animal studies providing a positive effect of the nanotubes on osteointegration. Further studies investigated the possible damaging effect of insertion into bone on nanotubular structures, which is another important aspect for the in vivo use in trauma and orthopedic surgery. Further, nanostructured surfaces might also be used for local drug delivery. The outlook goes beyond the scope of the review and discusses the impact of the COVID pandemic on the orthopedic field and the possibilities of smart implants equipped with different sensors to monitor healing.

From the 16 original publications reviewed in this mini-review, four used nanotubes to modify the surface and/or release drugs.

## 4. Discussion

The optimization of implants to improve osteointegration and reduce the risk of infection is still a strongly increasing current research field. Although total hip replacement was introduced over 100 years ago, it revolutionized orthopedic treatment in the 1960s and is therefore known as the “operation of the century” [35]; research still aims at the optimization of implant surface properties to improve osteointegration and infection prevention.

Due to increasing life expectancies and an active lifestyle also at older ages, implant technologies must be further developed to meet requirements such as early patient mobilization, implant longevity, infection-prophylaxis, and possibility for easy revision. The studies summarized in this mini-review represent the current approaches in trauma and orthopedic surgery utilized to meet these requirements: the modification of an implant materials’ surfaces for better osteointegration and anti-infective properties and the local drug release, as well as the combination of both. These approaches are not only used in orthopedic surgery, but also in craniofacial surgery as summarized in a review by Actis et al. [36].

The studies published in the first half of 2021 characterized the surface properties; the majority of the studies performed in vitro studies showing the effect on cells and bacteria, while only a few studies also proved the efficacy in animal models. To find their way to clinical application, these studies, however, are necessary.

The approaches using novel or modified materials to fight microbes are promising. However, some limitations should be mentioned: surface coatings or modifications of orthopedic and trauma implants must have a strong mechanical stability so that they withstand the insertion into the bone. Innovative medical devices and anti-infective strategies face several challenges, starting by the development, preclinical studies, entering and following the translational path to successful regulation and clinical use [16,37]. Limitations of the majority of the presented approaches are the complicated production of the surface modification and the necessity of a drug loading during production reducing the flexibility regarding the antimicrobial drug and increasing the manufacturing costs.

Future approaches might use a stimulus responsive approach to deliver a specific antibiotic or antimicrobial substance only when it is needed at a concentration sufficient to kill all bacteria. Ideally, the modification can be implemented on all implants and is stable against mechanical abrasion, which could occur during implantation of an intramedullary nail or a prosthesis. Controlled release and the stimulus responsiveness must be ensured to be effective and not cause resistant bacteria. Additive manufacturing, and more specifically 3D printing, could be an interesting approach to produce patient-specific antibacterial implants [38]. Antimicrobial peptides and ionic liquids could be interesting substances beside the classical antibiotics [39]. We expect that antibiotic-free antimicrobial biomaterials will play a greater role in the future, due to the inherent disadvantages of classic antibiotics such as resistance or toxicity. Such future antimicrobial biomaterials may for example include physical antimicrobial action. The antimicrobial approaches should be as simple as possible, not just to enable cost effective production, but also to meet the regulatory requirements. Effectiveness does not only have to be proven in controlled animal models, but also clinical data must show the reduction of infection in human clinical trials.

## 5. Conclusions

Material-associated infections are a feared complication in medicine, especially in trauma and orthopedic surgery, and are likely to increase in number due to antimicrobial resistance and a steadily growing number of implant operations as well as increased number of elderly immunocompromised patients. New material surface modifications and antimicrobial substances are researched to reduce the risk of infections. For translation into clinical applications, these approaches must be as simple as possible to allow manufacturing and meet regulatory requirements. The 16 original studies presented in this mini-review used quite different approaches and showed mostly promising results. However, the path to them being used to treat patients might be long and further studies are needed. Using novel materials-based strategies as alternatives and/or supplements to traditional antibiotic-based treatments of BAIs is an important and rapidly growing field in biomaterials science and medical science.

## Figures and Tables

**Table 1:** All publications found with the search strategy.

Year	1992–1995	1996–2000	2001–2005	2006–2010	2011–2015	2016–2020	2021
Paper #	3	14	23	57	110	190	18

**Table 2:** Summary of the 16 original studies.

*Modification*	*Original Studies on Materials, Methods, and Microbes; Published January–July 2021*				
**Surface modifications**	Effect of nano/microstructures on S. aureus adhesion on PET and titanium surfaces [17]	Impact of titanium surface modifications on biofilm development [18]	Enhanced antibacterial and osteogenic activity by hierarchically hybrid biocoatings [19]	Antibacterial and non-cytotoxicity properties of multi-scale hybrid modified coatings on titanium implants [20]	Porous metal-organic frameworks with synergistic antibacterial activity [21]
**Local antibiotic release**	Controlled release of gentamicin from dopamine-functionalized titanium surfaces coated with agarose-based hydrogels [22]	Antibiotic-loaded montmorillonite and poly-L-lysine-coating to reduce bacterial infections [23]	Antibiotic conjugated peptides coated on titanium as an infection-responsive antibacterial therapy [24]		
**Combination of both**	Titanium nanotube array/graphene nanocomposite coated with chitosan reveal improved biological characters [25]	Improved mammalian cells vs. bacterial colonization due to antimicrobial loading of nanotubular titanium [26]	Enhanced osteointegration and infection prevention realized by antibiotic-loaded amphora-shaped pores on titanium surface [27]		
**Non-metallic material modifications**	Antibiotic-loaded hydrogel for single-stage revision of MRSA orthopedic device-related infection [28]	Antibiotic-loaded hyaluronic acid hydrogel successfully eradicates chronic MRSA infection [29]	Pain relief and infection prevention by loaded chitosan membranes [30]	Synthesis and surface modification of peptide polymers for biocompatible antibacterial surfaces [31]	Silver nanoparticles coated nonwoven fabrics for skin wound healing [32]
